# Pre-dementia clinical trajectories associated with neuronal α-synuclein neuropathologic change: a retrospective cohort study

**DOI:** 10.21203/rs.3.rs-10788306/v1

**Published:** 2026-08-27

**Authors:** Elena Vera-Campuzano, Judit Selma-González, Íñigo Rodríguez-Baz, Carla Abdelnour, Federico Rodríguez-Porcel, Kate Wyman-Chick, Jon Toledo, Daniel Ferreira, Annegret Habich, Isabel Sala-Matavera, Julia Peris-Subiza, Jesús García-Castro, Sara Rubio-Guerra, Oriol Dols-Icardo, Olivia Belbin, Juan Fortea, Alberto Lleó, Daniel Alcolea, Ignacio Illán-Gala

**Affiliations:** 1 Sant Pau Memory Unit, IR SANT PAU, Hospital de la Santa Creu i Sant Pau, Barcelona 08025, Spain; 2 Centro de Investigación Biomédica en Red de Enfermedades Neurodegenerativas (CIBERNED), Madrid 28029, Spain; 3 Institut de Neurociències, Universitat Autònoma de Barcelona, Barcelona 08193, Spain; 4 Movement Disorders Division, Department of Neurology, Medical University of South Carolina, Charleston, SC, USA; 5 HealthPartners, Park Nicollet Struthers Parkinson’s Center, Golden Valley, Minnesota, Minneapolis, MN 55427, USA; 6 Nantz National Alzheimer Center, Stanley Appel Department of Neurology, Houston Methodist Hospital, Houston, TX 77030, USA; 7 Division of Clinical Geriatrics, Center for Alzheimer’s Research, Department of Neurobiology, Care Sciences, and Society, Karolinska Institutet, Stockholm 171 77, Sweden; 8 Facultad de Ciencias de la Salud, Universidad Fernando Pessoa Canarias, Santa María de Guía, Las Palmas 35450, Spain

## Abstract

Biological definitions of neuronal α-synuclein disease are advancing diagnosis beyond traditional clinical syndromes, but the trajectories preceding dementia and the influence of concomitant Alzheimer’s disease neuropathologic change (ADNC) remain uncertain. We analyzed longitudinal data from 1,543 participants without dementia at baseline who underwent repeated cognitive, functional, neuropsychiatric, and motor assessments and had neuropathological characterization at autopsy. Participants were classified as having neuronal α-synuclein neuropathologic change (NSNC), ADNC, or both. NSNC without intermediate or high ADNC was associated with the greatest motor burden. Among participants without mild cognitive impairment at baseline, concomitant ADNC was associated with earlier cognitive impairment and faster cognitive decline; mixed NSNC/ADNC also showed faster functional worsening than NSNC alone. Across participants with NSNC, baseline mild cognitive impairment, neuropsychiatric manifestations and concomitant ADNC predicted progression to dementia, whereas prespecified motor signs did not. These findings reveal heterogeneous pre-dementia trajectories and support combining α-synuclein detection with Alzheimer biomarkers, cognitive staging and multidomain clinical measures to improve prognosis and trial design.

## Introduction

The recently proposed neuronal α-synuclein disease (NSD) framework defines the disease biologically using biomarkers of neuronal α-synuclein and dopaminergic dysfunction, without requiring a specific clinical phenotype.^1^ At autopsy, the pathological substrate of NSD is neuronal α-synuclein neuropathologic change (NSNC). Clinical syndromes associated with NSNC map imperfectly onto the underlying pathology, particularly during prodromal or minimally symptomatic stages, when cognitive, neuropsychiatric, autonomic, sleep, and motor signs may be subtle, incomplete, and not yet associated with substantial functional impairment.^2^ Importantly, Alzheimer’s disease neuropathologic change (ADNC) frequently coexists with NSNC, modifying clinical phenotype and disease course.^3,4^ The coexistence of ADNC and NSNC has immediate translational relevance: anti-amyloid β (Aβ) therapies and emerging α-synuclein–targeted interventions require accurate attribution of clinical decline when ADNC and NSNC coexist.^5^

The increasing availability of biomarkers is accelerating the transition from syndrome-based diagnoses, such as Parkinson’s disease or dementia with Lewy bodies, towards biological definitions of disease.^1,6^ Aβ and tau biomarkers can identify or exclude ADNC in vivo, and α-synuclein seed amplification assays (SAA) now provide a biomarker-based approach to detecting NSNC.^7^ However, the longitudinal clinical trajectories associated with the earliest stages of NSD remain poorly characterized.^8,9^ Previous studies have generally started from established clinical syndromes, later-stage cohorts, or selected biomarker-enriched populations such as Parkinson’s disease or isolated REM sleep behavior disorder, potentially underrepresenting cognitive-first or neuropsychiatric-first presentations.^4,10–12^ To our knowledge, no previous large pathology-anchored study has jointly characterized cognitive, functional, neuropsychiatric, and motor trajectories before substantial functional impairment, while also examining how concomitant ADNC modifies these trajectories. Addressing this gap is important for selecting sensitive outcomes across the full spectrum of prodromal NSD features, when disease-modifying interventions are expected to be most effective.

Large, multicentre, autopsy-linked cohorts such as the National Alzheimer’s Coordinating Center (NACC) provide an opportunity to address this gap independently of clinical diagnostic labels. In this retrospective longitudinal autopsy study, we used repeated assessments obtained before substantial functional impairment to determine whether NSNC without intermediate or high ADNC, ADNC without NSNC, and mixed NSNC/ADNC were associated with distinguishable cognitive, functional, neuropsychiatric, and motor trajectories. Through this phenotype-agnostic, multidomain approach, aligned with the biological principles of the NSD framework, we aimed to characterize the early clinical course of NSNC, determine how ADNC modifies disease progression, and inform the selection of sensitive outcomes for early-stage NSD trials.

## Results

### Cohort characteristics and baseline profiles

The cohort included 1543 autopsy-confirmed participants: 150 with NSNC, 844 with ADNC, and 549 with NSNC/ADNC (**Supplementary Figure 1**; Table 1). Groups differed in age, sex, education, and APOE ε4 carriage. NSNC had the greatest first-visit motor burden, with higher UPDRS-III scores (5.7 vs 2.3 and 2.5; q=0.002) and more frequent moderate-to-severe substantia nigra hypopigmentation (36% vs 9% and 24%; q<0.001). Total NPI burden did not differ (q=0.13). **Supplementary Table 1** details co-pathologies.

At the first visit, MCI was present in 57 (38%) participants with NSNC, 359 (43%) with ADNC, and 296 (54%) with NSNC/ADNC (q<0.001). Among participants without MCI, memory performance was similar across groups, although language performance was lower in ADNC than in NSNC (q=0.03). Among participants with MCI, memory performance was lower in both ADNC and NSNC/ADNC than in NSNC (all q<0.001; **Supplementary Table 2 and Supplementary Figure 2**).

### Cognitive-functional stage transitions

**Figure 1.**
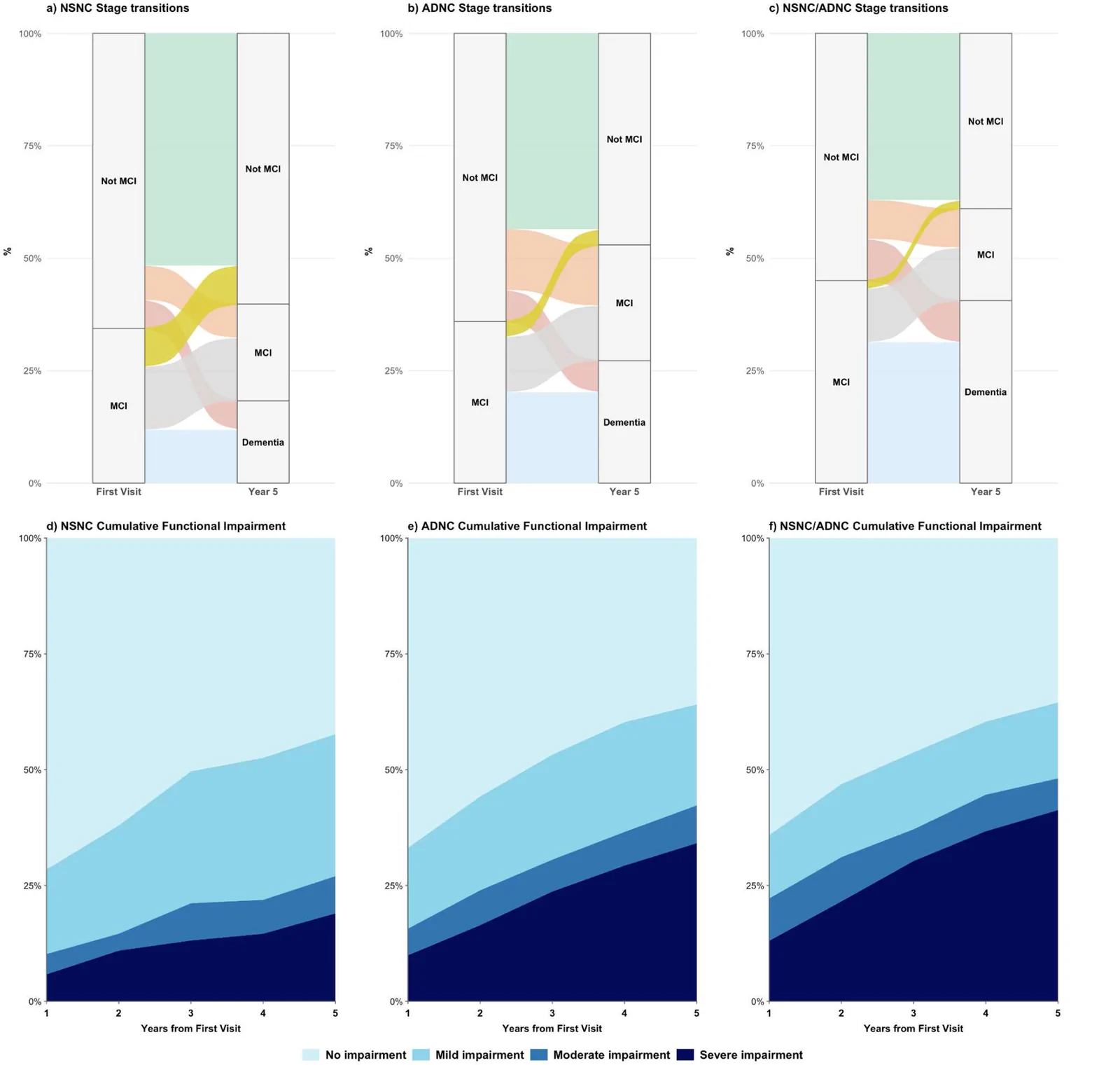
Cognitive-functional stage transitions and cumulative functional impairment by neuropathological group Panels a–c show cognitive-functional stage transitions from the first visit to the assessment conducted 5 years after baseline among the 946 of 1,543 participants (61.3%) with data available during this interval. Flow widths represent the percentage of participants within each neuropathological group. Panels d–f show the cumulative distribution of functional impairment, as measured by the FAQ, by each annual follow-up interval. At each visit, an item was considered affected when rated 1–3, and participants were classified as having no impairment (0 affected items), mild impairment (1–3 affected items), moderate impairment (4–5 affected items or at least two items rated 2), or severe impairment (≥6 affected items or at least two items rated 3). Abbreviations: NSNC, neuronal α-synuclein neuropathologic change; ADNC, Alzheimer’s disease neuropathologic change; NSNC/ADNC, neuronal α-synuclein neuropathologic change with concomitant Alzheimer’s disease neuropathologic change; FAQ, Functional Activities Questionnaire; Not MCI, no mild cognitive impairment; MCI, mild cognitive impairment.

Cognitive and functional stages diverged during follow-up (Figure 1). At the assessment conducted 5 years after the first visit, participants with NSNC were most likely to remain without MCI and least likely to have dementia, whereas NSNC/ADNC showed the greatest progression to dementia. Among participants without MCI initially, cognitive impairment at 5 years was present in 21% of NSNC and 32% of both ADNC and NSNC/ADNC. Among participants with MCI initially, 31% of those with NSNC, 56% with ADNC, and 70% with NSNC/ADNC reached the NACC-defined dementia stage, whereas reversion from MCI to Not MCI was more frequent in NSNC than in either ADNC-containing group (Figure 1).

Functional impairment accumulated in the same order, being lowest in NSNC and highest in NSNC/ADNC (Figure 1, panels d–f). By year 5, at least one cognitive, neuropsychiatric, or motor feature was present in 70% of participants with NSNC, 71% with ADNC, and 74% with NSNC/ADNC. Isolated motor positivity remained more frequent in NSNC than in ADNC or NSNC/ADNC (19% vs 10% and 6%; **Supplementary Figure 3**).

### Clinical profiles and exploratory neuropathological topography

Neuropsychiatric and motor profiles differed in specificity. Although total NPI burden was similar across groups, hallucinations and night-time behaviors showed the clearest enrichment in NSNC, particularly among participants with MCI (**Supplementary Figures 4 and 5**). Motor features differentiated the groups more consistently: most UPDRS-III domains were most prevalent in NSNC, and bradykinesia and axial signs affected 61–73% of participants with NSNC at the last assessment across first-visit cognitive strata (**Supplementary Figures 6 and 7**). Descriptive analyses by NSNC distribution suggested broader motor involvement with neocortical/diffuse NSNC, whereas brainstem-predominant NSNC was characterized particularly by bradykinetic and axial features (**Supplementary Figures 8 and 9**).

Topography-stratified analyses suggested that the prodromal course of NSNC depended on both its distribution and the presence of ADNC (**Supplementary Figures 10–12**). In the absence of ADNC, brainstem-predominant NSNC showed comparatively stable global and domain-specific cognitive trajectories and little progression of functional impairment, despite accumulating motor signs. Participants with brainstem-predominant NSNC and concomitant ADNC showed steeper cognitive and functional decline and were more frequently classified at the NACC-defined dementia stage at 5 years. ADNC was similarly associated with worse cognitive and functional trajectories in limbic/transitional and neocortical/diffuse NSNC.

**Figure 2.**
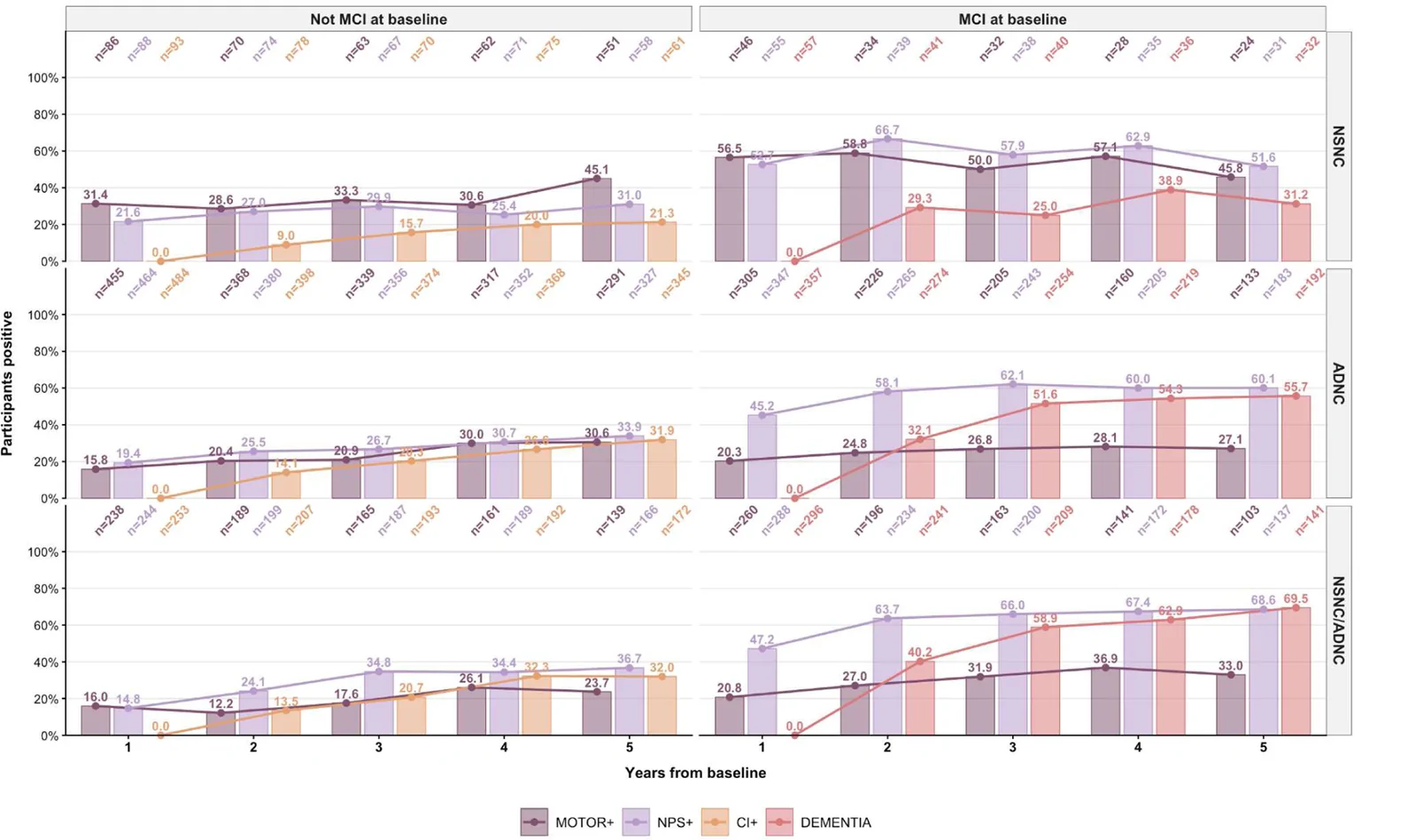
Prevalence of NSNC-related neuropsychiatric and motor changes and cognitive impairment by baseline cognitive status and neuropathological group Prevalence of NSNC-related neuropsychiatric and motor changes, cognitive impairment, and dementia across annual follow-up intervals during the first 5 years after baseline. Panels are stratified by baseline cognitive status (columns) and neuropathological group (rows). Bars and connected points represent the percentage of participants meeting each criterion within the corresponding follow-up interval. Percentage values are displayed above each bar, and color-coded n values indicate the number of participants with available data for each outcome and follow-up. Denominators represent participants with available data for the corresponding feature during the 5-year follow-up. Abbreviations: ADNC, Alzheimer’s disease neuropathologic change; CI+, cognitive impairment positivity; MCI, mild cognitive impairment; MOTOR+, NSNC-related motor changes; NPS+, NSNC-related neuropsychiatric changes; NSNC, neuronal α-synuclein neuropathologic change; NSNC/ADNC, concomitant neuronal α-synuclein and Alzheimer’s disease neuropathologic changes.

Figure 2 further illustrated the contrasting motor- and cognitive-weighted profiles across neuropathological groups. Among participants without MCI at the first visit, MOTOR+ at 5 years was more frequent in NSNC (45%) than in ADNC (31%) or NSNC/ADNC (24%), whereas cognitive impairment was less frequent in NSNC (21%) than in either ADNC-containing group (32% each). Among participants with MCI initially, neuropsychiatric positivity was common in all groups and, at 5 years, was present in 52% of NSNC, 61% of ADNC, and 69% of NSNC/ADNC; the proportion at the dementia stage was lowest in NSNC and highest in NSNC/ADNC (Figure 2).

### Longitudinal multidomain trajectories

**Figure 3.**
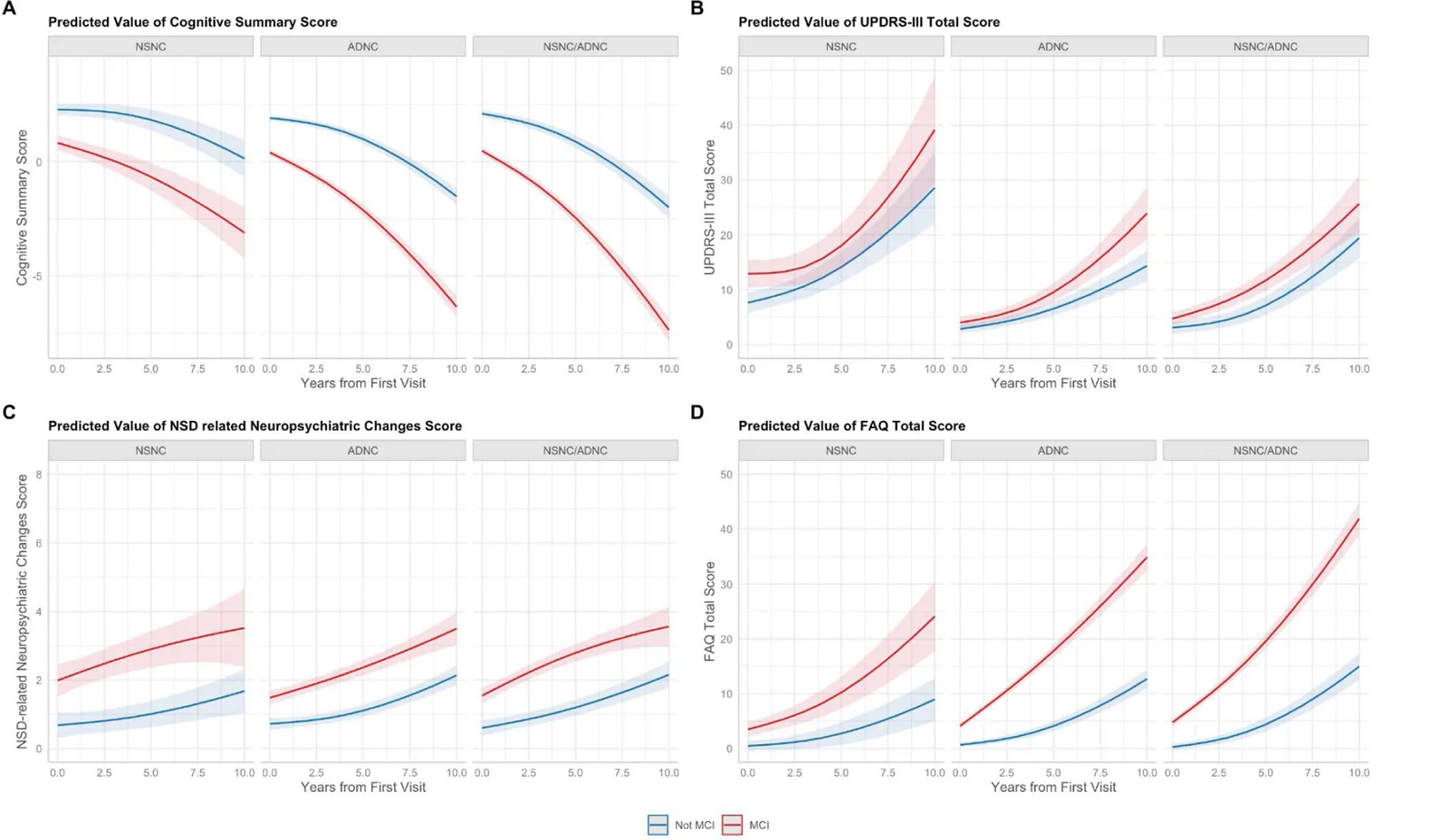
Adjusted longitudinal cognitive, motor, neuropsychiatric, and functional trajectories by neuropathological group and first-visit cognitive status Predicted trajectories of (A) CSS, (B) UPDRS-III total score, (C) NSD-related neuropsychiatric changes score, and (D) FAQ total score over 10 years, stratified by neuropathological group and first-visit cognitive status. Estimates were derived from complementary linear mixed-effects models using natural cubic splines for time and adjusted for age at first visit, sex, and education. Shaded bands represent 95% confidence intervals. Lower CSS values and higher UPDRS-III, neuropsychiatric, and FAQ scores indicate worse outcomes. Abbreviations: ADNC, Alzheimer’s disease neuropathologic change; CSS, Cognitive Summary Score; NSNC, neuronal α-synuclein neuropathologic change.

Linear mixed-effects models showed distinct longitudinal patterns of cognitive, motor, neuropsychiatric, and functional changes according to neuropathological group and first-visit cognitive status (Figure 3; **Supplementary Figure 13** and **Supplementary Tables 3**–**6**). Among participants without MCI at first visit, annual CSS decline was faster in ADNC than in NSNC (time × ADNC: β=−0.110, 95% CI −0.178 to −0.042; p=0.001) and in NSNC/ADNC than in NSNC (time × NSNC/ADNC: β=−0.172, −0.244 to −0.100; p<0.001). First-visit MCI was associated with faster CSS decline in NSNC (time × MCI: β=−0.138, −0.245 to −0.032; p=0.011), and this MCI-related difference was greater in NSNC/ADNC (three-way interaction: β=−0.128, −0.248 to −0.009; p=0.036); the corresponding interaction for ADNC did not reach statistical significance (p=0.059). Domain-specific models showed faster memory and language decline in both ADNC-containing groups among participants without MCI, whereas executive decline differed from NSNC only in NSNC/ADNC in this stratum (**Supplementary Tables 7**–**9**). Functional impairment increased faster in NSNC/ADNC than in NSNC among participants without MCI (time × NSNC/ADNC: β=0.520, 0.107 to 0.933; p=0.014), whereas the ADNC–NSNC difference was not significant (β=0.288, −0.097 to 0.674; p=0.142). First-visit MCI was associated with faster functional worsening in NSNC (time × MCI: β=0.834, 0.186 to 1.481; p=0.012), and this difference was greater in both ADNC (three-way interaction: β=0.973, 0.275 to 1.670; p=0.006) and NSNC/ADNC (β=1.053, 0.329 to 1.778; p=0.004). UPDRS-III burden was greatest in NSNC; among participants without MCI, motor worsening was faster in NSNC than in ADNC (time × ADNC: β=−0.675, −1.199 to −0.150; p=0.012) but did not differ significantly between NSNC and NSNC/ADNC (p=0.129). Motor slopes were not modified by first-visit MCI. Neuropsychiatric burden increased over time, without evidence that the rate of increase differed by neuropathological group or first-visit MCI (all longitudinal interaction p≥0.145). **Supplementary Tables 10**–**13** provide detailed topography-stratified mixed-effects model estimates.

### Progression to dementia

**Figure 4.**
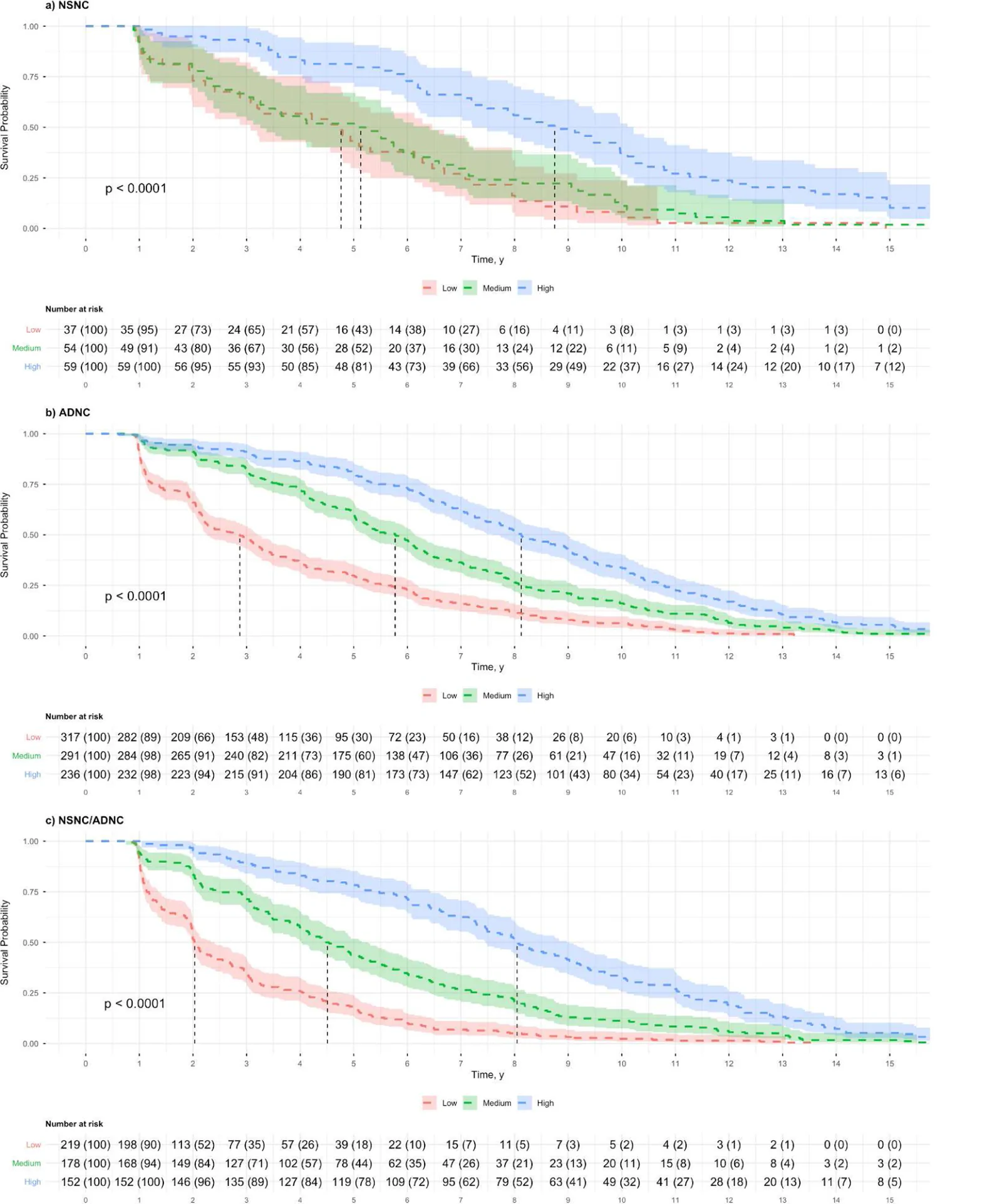
Time to the NACC-defined dementia stage by first-visit Cognitive Summary Score tertile Kaplan–Meier curves show dementia-free survival according to first-visit CSS tertile within each neuropathological group. Time was calculated from the first visit to the first visit at which the NACC-defined dementia stage was documented or the last available clinical assessment. Shaded areas represent 95% confidence intervals. P values were derived from log-rank tests. Vertical dashed lines indicate median dementia-free survival when reached. Lower CSS tertiles indicate poorer first-visit cognitive performance. Abbreviations: ADNC, Alzheimer’s disease neuropathologic change; CSS, Cognitive Summary Score; NSNC, neuronal α-synuclein neuropathologic change.

Lower first-visit CSS tertile was associated with progressively shorter time to the NACC-defined dementia stage in all neuropathological groups (all log-rank p<0.001; Figure 4). First-visit symptomatic status was also associated with shorter dementia-free survival (p<0.001 for NSNC and p<0.001 for ADNC and NSNC/ADNC; **Supplementary Figure 14**), with first-visit MCI providing the clearest prognostic separation (**Supplementary Figure 15**). In the multivariable analysis of participants in the NSNC and NSNC/ADNC groups, first-visit MCI showed the strongest association with progression (HR 2.82, 95% CI 2.34–3.39). NPS+ (HR 1.32, 95% CI 1.10–1.57; p=0.002), concomitant ADNC (HR 1.45, 95% CI 1.16–1.81; p=0.001), and APOE ε4 carriage (HR 1.22, 95% CI 1.02–1.46; p=0.029) were also independently associated with progression, whereas MOTOR+ was not (HR 1.06, 0.85–1.32; p=0.59; Table 2).

The primary findings were essentially unchanged after excluding the amygdala-predominant and olfactory-bulb-only subgroups (data not shown).

## Discussion

In this multicentre longitudinal autopsy study, we jointly characterized cognitive, functional, neuropsychiatric, and motor trajectories among participants with NSNC, ADNC, or mixed NSNC/ADNC, without selecting for a canonical clinical phenotype. Three findings stood out. First, among participants without MCI, NSNC without intermediate or high ADNC had the greatest motor burden and slower cognitive decline compared to both ADNC-containing groups; functional worsening was significantly faster only in mixed NSNC/ADNC. Second, first-visit cognitive status shaped subsequent course: in NSNC, MCI was associated with faster cognitive and functional worsening, with greater MCI-related functional decline in both ADNC-containing groups; first-visit MCI was also the strongest predictor of progression to the NACC-defined dementia stage. Third, NPS+ and concomitant ADNC independently predicted progression among participants with NSNC, whereas MOTOR+ did not. Exploratory analyses further suggested that brainstem-predominant NSNC without ADNC was associated with prominent motor deterioration and a more stable cognitive and functional course. Together, these findings distinguish features that enrich NSNC from those that predict cognitive-functional progression.

Previous clinicopathological studies established that the prodromal features of Parkinson’s disease and dementia with Lewy bodies are variable, and clinical-pathological correlations are imperfect. Executive or visuospatial impairment, REM sleep behavior disorder, subtle parkinsonism, fluctuations, and hallucinations can precede dementia, but no individual feature is obligatory, and amnestic or multidomain cognitive presentations are also common.^8^,^21^–^23^ Hallucinations can be relatively specific but are insufficiently frequent for broad early case identification, and a substantial proportion of pathology-confirmed cases lack parkinsonism or other core features at presentation.^8^,^23^ Our study extends this literature by jointly examining cognitive, functional, neuropsychiatric, and motor trajectories in a large phenotype-agnostic cohort with longitudinal follow-up and autopsy confirmation. The resulting profiles argue against a single obligatory motor-first or cognitive-first sequence as acknowledged by the NSD framework.

The topography-stratified analyses provide a descriptive explanation for this heterogeneity. In the absence of ADNC, brainstem-predominant NSNC was associated with accumulating bradykinetic and axial abnormalities but comparatively stable cognitive and functional trajectories, whereas more widespread NSNC was associated with broader motor involvement. This pattern is consistent with disease-progression modeling showing that early brainstem-predominant Lewy pathology is associated with a more motor-weighted course, whereas early limbic involvement is more closely associated with ADNC and poorer cognition.^9^ Our findings extend these observations by suggesting that NSNC distribution cannot be interpreted independently of ADNC: even brainstem-predominant NSNC was associated with a substantially worse cognitive-functional course when ADNC was present.

Across the primary analyses, ADNC was associated with faster cognitive decline, whereas mixed NSNC/ADNC showed the clearest functional acceleration; the topography-stratified findings were consistent with this pattern but remained exploratory. Previous clinicopathological studies have shown that ADNC (particularly tau burden) is associated with earlier dementia onset, shorter survival, and greater memory, language, and global cognitive impairment in Lewy body disease. 3,24–26 Using NACC autopsy data, Toledo and colleagues showed that neocortical Lewy pathology was preferentially associated with executive decline, whereas advanced tau pathology had a stronger influence on memory, language, and global cognitive progression.^25^ Biomarker-defined studies have similarly shown that individuals positive for both α-synuclein and AD biomarkers often have a more rapidly progressive and clinically ambiguous phenotype than those with Lewy pathology alone.^12^,^27^ Our study extends these observations by restricting the analysis to participants without dementia at first visit and by examining cognitive, functional, neuropsychiatric, and motor trajectories in parallel. The findings support the view that ADNC is a major correlate of the cognitive-functional expression of pre-dementia NSD.

The dissociation between pathological enrichment and cognitive-functional prognosis is also clinically relevant. Motor abnormalities differentiated the neuropathological groups more consistently than neuropsychiatric manifestations, yet the prespecified motor-positive profile did not independently predict progression to the NACC-defined dementia stage. Conversely, overall neuropsychiatric burden showed limited separation across neuropathological groups, but neuropsychiatric symptoms independently predicted progression among participants in the NSNC and NSNC/ADNC groups. Thus, the clinical features that help identify an NSNC-enriched phenotype are not necessarily those that best estimate proximity to cognitive-functional decline. Motor signs might identify a predominantly motor-weighted NSD trajectory without indicating high near-term dementia risk, whereas cognitive stage and neuropsychiatric manifestations might provide more direct prognostic information. This interpretation remains cautious because MOTOR+ was an operational measure rather than a biomarker of dopaminergic dysfunction, and its lack of association with progression to dementia does not imply that motor impairment is unimportant for mobility, independence, or quality of life.

These results are directly relevant to emerging biological frameworks for NSD.^1^,^6^ The NSD-ISS separates the biological definition of disease from its heterogeneous motor and non-motor features and recognizes the need to incorporate co-pathologies and functional anchors. Our findings support this phenotype-independent definition but also identify two dimensions that α-synuclein positivity alone does not resolve: pathological distribution and concomitant ADNC. In future biomarker-defined cohorts, α-synuclein seed amplification assays should therefore be interpreted alongside already available AD biomarkers, emerging positron emission tomography markers of neuronal α-synuclein, objective cognitive staging, and multidomain clinical measures. This approach might distinguish a relatively motor-weighted and cognitively slower NSD trajectory from an ADNC-modified trajectory with greater cognitive-functional risk. Finally, our study did not aim to formally validate or assign NSD-ISS stages because α-synuclein and dopaminergic biomarkers were unavailable; rather, it provides autopsy-grounded evidence on the clinical and mixed-pathology dimensions to consider when refining staging systems.

These distinctions are also important for therapeutic trials targeting α-synuclein. Selection based only on parkinsonism could exclude biologically affected participants with predominantly cognitive or neuropsychiatric manifestations, whereas enrichment based only on cognitive impairment could disproportionately select participants with ADNC. Trials targeting α-synuclein should therefore establish α-synuclein biology and prespecify stratification by AD biomarkers and baseline cognitive stage. The CSS detected differences in decline even among participants without MCI, whereas functional worsening was most evident in mixed NSNC/ADNC and was amplified by first-visit MCI. Cognitive and functional outcomes should therefore be selected and analysed according to baseline cognitive stage rather than assumed to be equally sensitive across prodromal NSD.^4^,^10^–^12^

The main strengths of this study are the large multicentre sample, neuropathological confirmation, restriction to participants without dementia at entry, long longitudinal follow-up, and parallel assessment of cognitive, neuropsychiatric, motor, and cognitive-functional outcomes. But the study is not without limitations. NACC is not population representative, and referral, survival, and consent-to-autopsy biases could affect the observed phenotypes. Neuropathology was determined at death, and it cannot establish when each pathological process became biologically detectable, and NSNC distribution at death cannot be assumed to represent its distribution or sequence during the earlier clinical assessments. NACC also incompletely captured fluctuations, REM sleep behavior disorder, autonomic dysfunction, olfaction, and detailed visuospatial performance. Finally, the motor and neuropsychiatric definitions were operational measures derived from available assessments and require validation in prospective biomarker-defined cohorts. Beyond APOE ε4 status, comprehensive genetic data were unavailable; GWAS data were available only for a subset of participants and would not have captured all pathogenic or risk variants considered in the NSD-ISS and SynNeurGe frameworks.^1^,^6^ This precluded genetic subclassification and assessment of genetic contributions to trajectory heterogeneity, but not the primary neuropathology-based classification.

In conclusion, pre-dementia NSD does not follow a single clinical sequence. NSNC without intermediate/high ADNC was predominantly motor-weighted and cognitively slower, whereas mixed NSNC/ADNC showed the steepest cognitive-functional course. Prospective biomarker-defined studies should integrate neuronal α-synuclein confirmation with AD biomarkers and multidomain clinical measures to stage disease and select sensitive trial outcomes.

## Methods

### Study population and data source

NACC collects standardized longitudinal clinical, neuropsychological, functional, behavioral, and neuropathological data from participants enrolled at current and past National Institute on Aging-funded Alzheimer’s Disease Research Centers (ADRCs) across the United States. At the time of data access, NACC included 37 active ADRCs; the present analysis included participants from 34 ADRCs. Participants are evaluated annually using the UDS protocol and may enroll in the brain donation program. For participants who consent, post-mortem neuropathological data are recorded using standardized NACC neuropathology forms and consensus criteria.

Demographic variables, clinical diagnoses, cognitive status, neuropsychological test scores, neuropsychiatric symptoms, motor signs, and functional measures were derived from the UDS. Neuropathological variables were derived from the NACC Neuropathology Data Set. The analysis used de-identified data from participants with longitudinal clinical assessments and autopsy-confirmed neuropathological information.

### Participants

Participants were eligible if they were aged 50 years or older; had autopsy data sufficient to classify ADNC and NSNC; had at least one eligible Cognitive Summary Score assessment; had no dementia at the first eligible cognitive assessment; and had at least two cognitive assessments. Progression to a specific clinical stage or syndrome was not required. Participants were then restricted to those meeting neuropathological criteria for ADNC, NSNC, or both. Baseline corresponded to the first eligible cognitive assessment and the last visit to the final available cognitive assessment.

### Detailed neuropathological classification

ADNC and NSNC were coded independently to classify biologically relevant proteinopathies rather than predict a specific phenotype, consistent with recent biological frameworks for NSD. The intermediate/high ADNC threshold was used as an operational proxy for a burden likely to contribute to clinical trajectories and broadly aligned with the level of AD pathology targeted by contemporary amyloid and tau biomarker frameworks.^13^

Participants were assigned to three mutually exclusive groups: NSNC (NSNC positive, ADNC negative), ADNC (ADNC positive, NSNC negative), or NSNC/ADNC (both positive).

NSNC distribution was coded using standardized NACC categories: brainstem-predominant, limbic/transitional, neocortical/diffuse, amygdala-predominant, and olfactory-bulb-only. These categories were treated as topographical descriptors. Amygdala-predominant and olfactory-bulb-only patterns were included in the primary analysis but excluded in the sensitivity analysis because their relation to clinical trajectories is less certain and they may be detected with lower sensitivity by currently available α-synuclein seed amplification assays.^14,15^

Vascular brain injury, cerebral amyloid angiopathy, hippocampal sclerosis, limbic-predominant age-related TDP-43 encephalopathy neuropathologic change (LATE-NC^16^), and primary age-related tauopathy (PART^17^) were summarized descriptively but did not define the three primary neuropathological groups.

### Characterization of cognitive changes

At the first visit, participants were classified according to the NACC clinician consensus cognitive status diagnosis as not-MCI (normal cognition or impaired-not-MCI) or mild cognitive impairment (MCI). Harmonized memory, language, and executive-function composites were generated through confirmatory factor analysis using anchor items to place scores on a common scale across cohorts and time points, as previously described.^18^ Because NSD-related cognitive impairment can involve multiple domains, we combined the three domain scores into a Cognitive Summary Score (CSS) to capture change across the cognitive profile rather than rely on any single domain. Studies in Parkinson’s disease support this approach, showing that cognitive composites can improve sensitivity to early cognitive differences and longitudinal decline relative to individual tests or brief global measures.^19,20^ The CSS was calculated as the unweighted sum of the memory, language, and executive-function composites and only when all three component scores were available; higher scores indicated better cognitive performance. The Mini-Mental State Examination (MMSE) and the CDR^®^ Dementia Staging Instrument (CDR) were also included as general measures of cognitive and functional impairment.^21^

### Characterization of neuropsychiatric changes

Neuropsychiatric symptoms were assessed using the Neuropsychiatric Inventory (NPI). Participants were considered positive for NSNC-related neuropsychiatric changes (NPS+) if at least one of the following NPI items was endorsed at two consecutive visits: hallucinations or delusions, apathy, depression or anxiety, or nighttime behaviors. The selection of NPI domains was prespecified from prior literature.^22,23^ The persistence requirement was chosen to prioritize sustained behavioral change over single-visit symptoms.^24,25^

### Characterization of motor changes

Motor signs were assessed using the Unified Parkinson’s Disease Rating Scale Part III (UPDRS-III). Operational motor positivity (MOTOR+) was defined by a UPDRS-III total score ≥5 together with at least one abnormal akinetic-rigid or hypomimic feature: facial expression/hypomimia, rigidity, or bradykinesia. This definition was chosen based on previous evidence suggesting that akinetic-rigid signs and hypomimia are more closely related to dopamine transporter abnormality than the UPDRS-III total score alone ^26^, and that they also track Lewy body pathology in autopsy-confirmed cohorts.^27^ A total UPDRS-III score threshold of 5 points was intended to capture subtle motor involvement that may already accompany dopamine transporter abnormality.^28–30^ For domain-level analyses, UPDRS-III motor items were binarized and grouped into speech, facial expression, tremor at rest, action/postural tremor, rigidity, bradykinesia, and axial motor features, as in previous studies.^31^ Each domain was coded as present when at least one contributing item was abnormal; domains with all contributing items missing were coded as missing.

### First-visit symptomatic status

For analyses of first-visit symptomatic status and dementia-free survival, neuropsychiatric positivity was based on the first visit only; the two-visit persistence criterion was applied only to longitudinal NPS+ analyses. Participants were classified as symptomatic if they had MCI, NPS+, or the prespecified akinetic-rigid/hypomimic MOTOR+ profile at the first visit. Participants without any of these features were classified as asymptomatic.

### Characterization of functional impairment

Functional impairment was assessed using the 10-item Functional Activities Questionnaire (FAQ). At each visit, an item was considered affected when rated 1–3, and participants were classified as having no impairment (0 affected items), mild impairment (1–3 affected items), moderate impairment (4–5 affected items or at least two items rated 2), or severe impairment (≥6 affected items or at least two items rated 3). A single item rated 3 was recorded as a focal dependency but did not automatically determine the severe stage. These criteria adapt the NACC deficit-count framework proposed by Devanand and colleagues while incorporating item-level severity.^32^

### Outcome hierarchy

The primary longitudinal outcome was rate of change in the CSS. Secondary longitudinal outcomes were FAQ total score, UPDRS-III total score, and the NSD-enriched neuropsychiatric changes score. Cognitive-functional stage transitions and progression to the NACC-defined dementia stage were secondary descriptive and prognostic analyses, respectively. Memory, language, and executive-function trajectories, symptom-domain profiles, and analyses stratified by NSNC topography were exploratory. Exclusion of participants with NSNC restricted to the amygdala or olfactory bulb was considered a sensitivity analysis.

### Handling of missing data

NPI, UPDRS-III, and FAQ total scores were calculated as unprorated sums of available constituent items and set to missing only when all constituent items were unavailable. No imputation was performed. Linear mixed-effects models used all available repeated outcome observations and model-specific complete covariate data; Cox models used complete cases for the prespecified covariates.

### Statistical analysis

Analyses were performed in R version 4.5.1. Continuous variables were summarised as mean (SD) when approximately symmetric and as median (IQR) when markedly skewed. Categorical variables were summarised as n (%). Between-group differences across the three neuropathological groups were assessed using the Kruskal–Wallis test for continuous variables and the χ^2^ test or Fisher’s exact test for categorical variables. Pairwise post hoc comparisons were performed when omnibus tests were significant. Benjamini–Hochberg correction was applied separately within each family of pairwise comparisons: first-visit demographic and clinical variables, neuropsychiatric domains, and motor domains. For transition analyses, longitudinal cognitive status (Not MCI, MCI, or Dementia) was evaluated from the first visit to the last available clinical visit. Domain-level prevalence was calculated for each group and displayed using radar plots and heatmaps. Neuropsychiatric and motor symptom profiles were compared descriptively across neuropathological groups and cognitive strata at the first visit and last follow-up. Group differences in symptom prevalence were assessed using χ^2^ or Fisher’s exact tests, with post-hoc pairwise comparisons when appropriate.

Primary linear mixed-effects models included fixed effects for time, neuropathological group, first-visit cognitive status, age, sex, and education, together with all two-way and three-way interactions among time, neuropathological group, and first-visit cognitive status. Participant-specific random intercepts and slopes for time were included. Given the observed nonlinear trajectory of cognitive outcomes over long follow-up periods, complementary models replaced the linear time term with a natural cubic spline with two degrees of freedom.^33^ These models were used to display potentially nonlinear predicted trajectories. Predictions were restricted to the first 10 years of follow-up because data were sparse thereafter.

Kaplan–Meier curves were used to describe time to the NACC-defined dementia stage according to first-visit CSS tertile within each neuropathological group; tertiles were used for graphical presentation only. Event time was assigned to the first visit at which NACC-defined dementia was documented, and participants without progression were censored at their last clinical assessment.

The multivariable prognostic analysis was restricted to participants in the NSNC and NSNC/ADNC groups. The Cox model included concomitant ADNC, first-visit MCI status, APOE ε4 carrier status, NPS+ using only first-visit data, MOTOR+, age, sex, and education. Covariates were selected a priori and entered simultaneously. Hazard ratios are reported with 95% confidence intervals. The proportional-hazards assumption was assessed using scaled Schoenfeld residuals, and continuous-covariate functional forms were evaluated graphically. Robust standard errors accounted for clustering at the ADRC level. The analytical sample size, number of events, and number excluded because of missing covariates were reported with the model results.

All tests were two-sided. A p value below 0.05 was considered statistically significant for the primary analyses; secondary and exploratory analyses were interpreted with consideration of multiplicity.

### Ethics

Written informed consent was obtained from participants and study partners at each participating ADRC. Institutional review board approval was obtained at each center for collecting clinical and neuropathological data and for submitting de-identified data to NACC. The present study used de-identified data and was conducted in accordance with NACC data-use agreements.

### Use of artificial intelligence

The authors used ChatGPT (OpenAI) to improve the clarity and scientific writing of the manuscript. The authors developed all scientific ideas, hypotheses, methods, analyses, interpretations, and conclusions. The authors reviewed and edited all AI-assisted text and take full responsibility for the manuscripťs content.

## Supplementary Material

Supplementary Files

This is a list of supplementary files associated with this preprint. Click to download.

• NatureCommunicationsLBDSupplementary.pdf

## Figures and Tables

**Table 1.** First-visit demographic and clinical characteristics by neuropathological group.

Characteristic	NSNC n = 150	ADNC n = 844	NSNC/ADNC n = 549	p value	q value
Age at baseline	78 (7)	78 (8)	76 (8)	<0.001	<0.001
**Biological sex**				<0.001	<0.001
Female	67 (45%)	484 (57%)	256 (47%)		
Years of education	17 (3)	16 (3)	16 (3)	0.009	0.014
**APOE ε4 status**				<0.001	<0.001
Carrier	32 (21%)	397 (47%)	306 (56%)		
Mini-Mental State Examination	29 (2)	28 (2)	28 (2)	<0.001	0.001
Functional Activities Questionnaire	1.3 (2.8)	1.8 (3.8)	2.3 (4.3)	0.2	0.2
CDR Sum of Boxes	0.69 (1.06)	0.78 (1.09)	0.99 (1.21)	<0.001	<0.001
UPDRS-III total score	5.7 (9.0)	2.3 (4.3)	2.5 (5.1)	0.001	0.002
NPI total score	1.7 (3.3)	1.4 (2.4)	1.7 (2.7)	0.10	0.13
**Cognitive status**				<0.001	<0.001
MCI	57 (38%)	359 (43%)	296 (54%)		
Time from last visit to death	3.2 (2.9)	3.3 (2.8)	3.7 (2.8)	0.005	0.007
Number of visits	7 (3)	7 (3)	6 (3)	0.2	0.2
Mean follow-up time	6.2 (3.6)	6.0 (3.4)	5.7 (3.3)	0.15	0.2
**Substantia nigra hypopigmentation**				<0.001	<0.001
None–mild	93 (64%)	737 (91%)	396 (76%)		
Moderate–severe	53 (36%)	73 (9%)	126 (24%)		

Values are mean (SD) or n (%), unless otherwise indicated. Between-group comparisons across neuropathological groups used Kruskal–Wallis tests for continuous variables and Pearson’s χ^2^ or Fisher’s exact tests for categorical variables. P values were adjusted for multiple comparisons using the Benjamini–Hochberg false discovery rate procedure. NPI, UPDRS-III, and FAQ total scores were calculated from available items and set to missing when all constituent items were unavailable; for the NSNC, ADNC, and NSNC/ADNC groups, respectively, missing data were 9/150 (6%), 55/844 (7%), and 28/549 (5%) for NPI; 18/150 (12%), 87/844 (10%), and 53/549 (10%) for UPDRS-III; and 38/150 (25%), 219/844 (26%), and 155/549 (28%) for FAQ.

Abbreviations: ADNC, Alzheimer’s disease neuropathologic change; APOE, apolipoprotein E; CDR-SB, Clinical Dementia Rating Sum of Boxes; FDR, false discovery rate; MCI, mild cognitive impairment; MMSE, Mini-Mental State Examination; NACC, National Alzheimer’s Coordinating Center; NPI, Neuropsychiatric Inventory; NSNC, neuronal α-synuclein neuropathologic change; SD, standard deviation; UPDRS-III, Unified Parkinson’s Disease Rating Scale Part III.

**Table 2.** Multivariable Cox proportional hazards model for progression to the NACC-defined dementia stage among participants in the NSNC and NSNC/ADNC groups.

Variable	HR	SE	CI low	CI high	z	p
Age at baseline	1.005	0.006	0.993	1.017	0.799	0.424
Male	1.084	0.095	0.912	1.288	0.916	0.360
Education	0.991	0.015	0.962	1.021	−0.592	0.554
APOE ε4 carrier	1.221	0.112	1.021	1.461	2.188	0.029
Concomitant ADNC	1.448	0.163	1.161	1.805	3.289	0.001
MCI at first visit	2.816	0.268	2.337	3.393	10.885	<0.001
NPS+ at first visit	1.318	0.119	1.104	1.573	3.051	0.002
MOTOR+ at first visit	1.062	0.117	0.856	1.317	0.543	0.587

The analysis included participants from the NSNC and NSNC/ADNC groups (n=699). Hazard ratios were estimated using a multivariable Cox proportional hazards model, with progression to dementia as the event and time from the first visit to the first visit at which the NACC-defined dementia stage was documented or the last available clinical assessment as the timescale. The model included neuropathological group, first-visit cognitive status, APOE ε4 carrier status, NPS+, MOTOR+, age at first visit, sex, and education. Reference categories were female sex, no MCI at the first visit, absence of NPS+ at the first visit, absence of MOTOR+, absence of concomitant ADNC, and APOE ε4 non-carrier status. HR >1 indicates a higher risk of progression to dementia. The complete-case model included 569 of 699 eligible participants; 130 participants were excluded because of missing covariate data.

Abbreviations: ADNC, Alzheimer’s disease neuropathologic change; APOE, apolipoprotein E; CI, confidence interval; HR, hazard ratio; MCI, mild cognitive impairment; NPS+, neuropsychiatric positivity; NSNC, neuronal α-synuclein neuropathologic change; MOTOR+, motor positivity.

## Data Availability

Deidentified participant-level data used in this study were obtained from NACC and cannot be redistributed by the authors. Researchers can apply for access directly through the NACC data-request process, subject to NACC eligibility requirements and execution of the applicable data-use agreement.

## Code availability

The study analytic code will be shared by the corresponding author upon reasonable request.
